# Low-Dose Quetiapine Causing Agranulocytosis and Leucopenia in a Patient with Benign Neutropenia: A Case Report

**DOI:** 10.7759/cureus.8505

**Published:** 2020-06-08

**Authors:** Vincent Fryer, James Billings

**Affiliations:** 1 Northeastern Wisconsin Psychiatry Program, Medical College of Wisconsin Affiliated Hospitals, Green Bay, USA

**Keywords:** quetiapine, agranulocytosis, leucopenia, depression

## Abstract

Low white blood cell (WBC) counts and agranulocytosis are a relatively rare side effect of atypical antipsychotic treatment. Often, quetiapine is used as an adjunctive medication to help with sleep and mood in some patients. Many times, it is prescribed at lower doses when targeting this mechanism of action, especially when used in combination with antidepressants. The following case highlights the importance of understanding the potential risks in prescribing quetiapine in patients who have underlying genetic susceptibility towards low white blood cell counts.

## Introduction

Quetiapine was approved for medical use in the United States in 1997. It is approved in the US for the treatment of schizophrenia, bipolar depression, and as an augmenting agent to serotonin specific reuptake inhibitors (SSRIs)/serotonin norepinephrine reuptake inhibitors (SNRIs) in unipolar depression. It is also commonly used in multiple other settings as off-label use.

It is associated with a unique clinical profile similar to clozapine, with an efficacy similar to that of other atypical antipsychotics in the augmentation of the treatment of unipolar depression [1-2]. Quetiapine is used with a distinct dosing strategy, which corresponds to its receptor occupancy at differing dosages. At doses of approximately 25-200 mg per day, it exhibits primarily H1 antihistaminic properties. As such, it is primarily used as a hypnotic for sleep [3-4]. With doses around 300-600 mg per day, quetiapine is primarily a 5HT2A-D2 antagonist with adjunctive antihistaminic properties [3,5]. At this dosing range, quetiapine is used for its antidepressant actions. At doses of 700-800 mg per day, there is a high saturation of H1, 5HT2A, and D2 receptors; as such, this is primarily used for its antipsychotic properties [3-4].

Quetiapine, like other atypical antipsychotics, has similar side effects common to the class of medications, including weight gain, hypercholesteremia, and QTC prolongation [3]. However, it is rather unique in its sedation properties, which occur at low doses [3-4]. Quetiapine, like most atypical antipsychotics, only has a 1%-4% risk of low blood cell counts [5].

## Case presentation

This the case of a 35-year-old white male who was hospitalized under an involuntary hold for suicidal ideation. The patient endorsed he had taken quetiapine 50 mg per day consistently for the past five months for insomnia symptoms primarily. The patient had previously trialed trazodone and hydroxyzine, with a poor response. The patient previously had borderline low WBC counts for approximately 15 years according to his personal knowledge. To his recollection, he was first diagnosed with benign neutropenia while incarcerated some 15 years prior. Records from his previous incarceration were not available during his hospitalization. The patient reported that his WBC counts were in the range of 2000-4000 WBCs per microliter. 

While hospitalized, his outpatient medication regimen of quetiapine 50 mg per day for his depression and insomnia symptoms was continued. This resulted in a decrease in his depression and suicidal ideation and in improvement in sleep.

On admission, his WBC count was 1200 WBC per microliter. His absolute neutrophil count was 996 WBCs. All other lab values that were drawn were within normal limits. The patient was given prophylactic antibiotics for this and remained stable with no infection or fever noted during his hospital course. After an investigation into the cause of his neutropenia and agranulocytosis, his quetiapine was discontinued, as it was believed to be the causative agent.

This discontinuation resulted in a return to baseline WBC counts for the patient after two weeks free from the quetiapine. Subsequently, the patient was discharged from hospital on mirtazapine with low levels of depressive symptoms and suicidal ideation reported.

## Discussion

Quetiapine is commonly used as off-label use as a second or third-line medication when patients have previously trialed other sleeping medications [4,6]. It also seems to be a reasonable choice given the patient's previous trials of medications and current depressive symptoms [1-2,7].

Common side effects with quetiapine increase in a dose-response fashion, given the medication's relative binding affinities at different dosages [3]. According to the manufacturers of risperidone and paliperidone, they have approximate rates of 4% and 2%, respectively, for neutropenia [8-9]. The manufacturers of quetiapine state that its risk is approximately 2% [10].

In a literature review of 21 cases of leukopenia, neutropenia, and thrombocytopenia caused by quetiapine, only one case, with a daily regimen of 50 mg of quetiapine, caused clinically significant low WBCs. However, in that case, there were multiple other medications used and the patient had a diagnosis of Parkinson’s disease [11]. The majority of cases reported in this literature review were using quetiapine and valproate combination therapy. In all 21 cases, multiple medications were simultaneously being used in the patients’ regimen [11].

When looking into reasons for this phenomenon, there was evidence that that cytochrome P450 2D6 catalyzes the formation of 7-hydroxyquetiapine. This forms a reactive quinone-imine and a reactive radical, which may account for the neutrophil toxicity. This was also replicated in vitro with olanzapine [12]. This mechanism is said to be the reason that the rates of blood dyscrasia are lower for quetiapine than that of clozapine, even though they have a similar chemical structure. However, in a meta-analysis, it was found that clozapine has no stronger association with neutropenia than other antipsychotic medications [13]. Rather, this may be a class-related effect. Other cases have postulated that quetiapine may have a dose-response relationship relating to its propensity to causing leucopenia [14].

In other literature and case reports, there have been cases of patients who developed antipsychotic-induced neutropenia on one medication and subsequently developed neutropenia when taking other antipsychotics [15]. There may be a possible sensitization to the development of antipsychotic-induced neutropenia. This patient population may also have an underlying susceptibility to low WBCs. This question requires further investigation and research.

Our case is distinct in that neutropenia (leukopenia) was induced by quetiapine monotherapy at a dose of 50 mg per day, and the patient also did not have a diagnosis of bipolar disorder or schizophrenia. It is postulated that in our case that the cumulative effects of the 50 mg of quetiapine over five months resulted in this, however, further research in the future will be required. Another explanation may be that the patients’ benign neutropenia diagnosis predisposed him towards low WBC counts. In a retrospective study where antipsychotics were administered in patients with benign neutropenia, there was no decrease in neutrophil counts and all cases maintained a stable neutrophil count [16]. Most cases of leukopenia and agranulocytosis with quetiapine occur with doses above 200 mg per day and multiple medications and conditions [11].

## Conclusions

This case highlights the importance of understanding antipsychotic medications and their effects on the hematological system, especially in patients with familial neutropenia. The absence of physical symptoms to alert both clinicians and patients to immunological compromise jeopardizes the timely identification of potentially marked neutropenia. Mental health providers should consider the risks of low WBCs when prescribing quetiapine, especially in patients with underlying hematological abnormalities.
